# Exploring the Potential Consortium of Migraine and Periodontitis

**DOI:** 10.1155/2024/3559500

**Published:** 2024-04-25

**Authors:** Deepa Ponnaiyan, Roshan R. Rughwani, Ganesh Shetty, Jaideep Mahendra, Dhayanand John Victor, Kaustubh Suresh Thakare, N. Sowmya Reddy

**Affiliations:** ^1^SRM Dental College, Chennai, Tamil Nadu, India; ^2^Shyam's Namma Dentist Dental Clinic, Royapettah, Chennai, India; ^3^Dental and Orthodontic Clinic, Bangalore, India; ^4^Meenakshi Academy of Higher Education and Research, Chennai, India; ^5^VYWS Dental College and Hospital, Amravati, India

## Abstract

**Objectives:**

Various researches have shown periodontitis to share common pathophysiological pathways with systemic diseases such as diabetes, cardiovascular diseases, and osteoporosis and recently neurological disorders. This article provides a narrative review summarizing the various linking mechanisms and the nature of association between two multifactorial diseases—periodontitis and migraine.

**Materials and Methods:**

A literature search was performed for articles related to periodontitis and migraine up till the year 2023 which yielded totally 14 articles. There were only three randomized controlled clinical trials; therefore, we were unable to conduct a systematic review and focused on a narrative review. The keywords searched were “migraine”, “periodontitis” and “biomarkers” in PubMed/Medline, Web of Science, and Embase databases. Any article related to the association of periodontitis and migraine and the dental management of subjects with headache disorders were included and studies with migraine and other dental diseases were excluded.

**Results:**

It is found that the occurrence of periodontitis and migraine are associated with each other. There is reasonable evidence to believe that periodontitis and migraine are linked by direct and indirect mechanisms which can eventually lead to chronic inflammatory conditions like periodontitis worsening neurovascular conditions such as migraine. However, upon detailed analysis it was found that the strength of association is weak owing to the presences of various common confounding and risk factors.

**Conclusions:**

The association between periodontitis and migraine cannot be denied, however, not all the criteria are fulfilled while examining the nature of association and future long-term studies are required to prove the same. *Clinical Relevance*. Various studies have reported poor periodontal health in patients with migraine. The risk of exacerbation of migraine also increases in subject undergoing dental therapy if the triggering factors are manipulated. Hence, knowing the precise pathophysiologic mechanisms linking both the diseases would be favorable in planning treatment protocols for subjects with migraine.

## 1. Introduction

Periodontitis is a multifactorial inflammatory disease of the tooth-supporting structures that is initiated due to the host response in retaliation to the biofilm accumulated on the hard nonshedding surfaces of the oral cavity [1]. The disease is characterized by an altered immune response to the invading microorganisms that causes the release of various proinflammatory cytokines and chemokines in addition to the depletion of the proresolving mediators which causes the periodontium to breakdown at the macroscopic level [2, 3]. The proinflammatory mediators, the bacteria and the bacterial by-products have been additionally known to spill into the systemic circulation which triggers the disease initiation at anatomically distinct sites which further supports the “focal infection theory” and lays the bedrock for research in the field of periodontal medicine [4].

Periodontitis, which is the sixth most common disease to occur in humans, is known to be associated with conditions such as diabetes, cardiovascular diseases, rheumatoid arthritis, osteoporosis, male and female reproductive pathologies, and also neurological diseases such as Alzheimer's, Parkinson's disease, and migraine [5].

Migraine, which accounts to about 14.7% of all neurological disorders, is characterized by unilateral pulsatile headache which may or may not occur in unison with a migrainous aura [6]. The aura, is characterized by flashes of light and visual disturbances followed by deep headache upon activation of the trigeminovascular pain pathway due to various trigger factors [7]. Migraines maybe classified as primary or secondary wherein the headache in the primary migraine is precipitated by neurologic causes, without any other underlying cause. Secondary migraine is a resultant of pathologies involving the head and neck, substance abuse, vascular disorders, psychiatric disorders, and also infections of the cranium, eyes, ear, nose, teeth, and its supporting structures [8]. However, secondary migraine has been associated with various trigger factors such as stress, hormonal disturbances, alcoholism, weather changes, sensory stimuli, and also smoking [9, 10]. It is an evident fact that smoking and various other risk factors such as stress may have a role to play in common pathophysiological pathways orchestrating the occurrence of both periodontitis and migraine. Hence, the current review will elaborate on the effect of periodontitis on migraine and trying to examine the common pathophysiological pathways and the current understanding of the association between the two diseases. Recent systematic reviews have tried to assess the association of migraine and periodontitis by studying the role of pro-inflammatory marker elevation and their role in migraine; however, there have been no reviews that have focused on the various other etiological factors in the pathophysiology of migraine and periodontitis. Therefore, the current review includes a detailed understanding of the various direct and indirect mechanisms that can orchestrate the possibility of occurrence of both the diseases. Further, the evidence on dental treatment protocols for migraineurs is also sparce and we have discussed the same in our review.

## 2. Method of Searching the Available Literature

The literature evidence was searched from inception till 2023 using MeSH keyword such as “periodontitis”, “migraine”, and “biomarkers”. Fourteen articles were retrieved via electronic sources (11-Pubmed; 1- Scopus; 1-Google Scholar, Embase, and WOS). Three articles were randomized controlled trials and one was a longitudinal study and the rest 10 were reviews. Since there was very sparse literature evidence, none of the data were excluded in our narrative review which is also a reason as to why we were unable to conduct a systematic review on our topic of discussion—migraine and periodontitis.

## 3. Mechanisms Linking Migraine and Periodontitis

**Figure 1:**
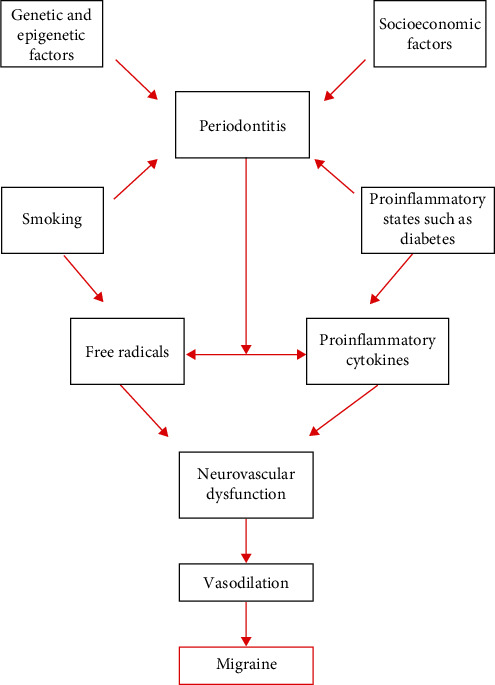
Pathophysiological pathway linking periodontitis with migraine. The flowchart illustrates the various risk factors that cause periodontitis. The systemic burden of periodontitis eventually is associated with the occurrence of migraine as the various chemical mediators cause neurovascular dysfunction.

Migraine and periodontitis share common risk factors however, the plausibility of these risk factors to cause both the diseases simultaneously is of great interest and maybe explained through direct and indirect pathophysiological pathways (Figure 1).

### 3.1. Direct Pathophysiological Mechanism

Periodontitis is characterized by an altered microbial flora in deep periodontal pockets. The primary pathogens causing periodontal disease are *P. gingivalis*, *T. denticola*, *Tanellera forsythia*, and *Aggegagatibacter actimocytemcomitans* which gain access through the ulcerated periodontal tissues and open the gateway for the entry of these microorganisms into the vasculature that may get carried to anatomically unrelated sites where they may get lodged and cause disease initiation [11]. Alternatively, the inherent capacity of keystone periodontal pathogen, *P. gingivalis* to thrive intracellularly within the inflammatory cells and get transported to distant tissues forms the basis of “Trojan Horse approach” of direct bacterial spread [12]. It is interesting to know that *P. gingivalis* is positively associated with migraine in older Hispanic women after adjusting for confounders [13]. Additionally, less commonly known periodontal pathogens such as *H. pylori* that has traditionally been associated with gastrointestinal disturbances, is also linked with the occurrence of migraine as studied in a meta-analysis [14]. Since, the oral biofilm is also a reservoir of *H. pylori*, the association of elevated *H. pylori* numbers with the occurrence of chronic periodontitis cannot be negated [15]. Thus, direct spread of bacteria or dysbiosis leading can lead to significant neurovascular changes and lead to migraine.

### 3.2. Indirect Mechanisms

**Figure 2:**
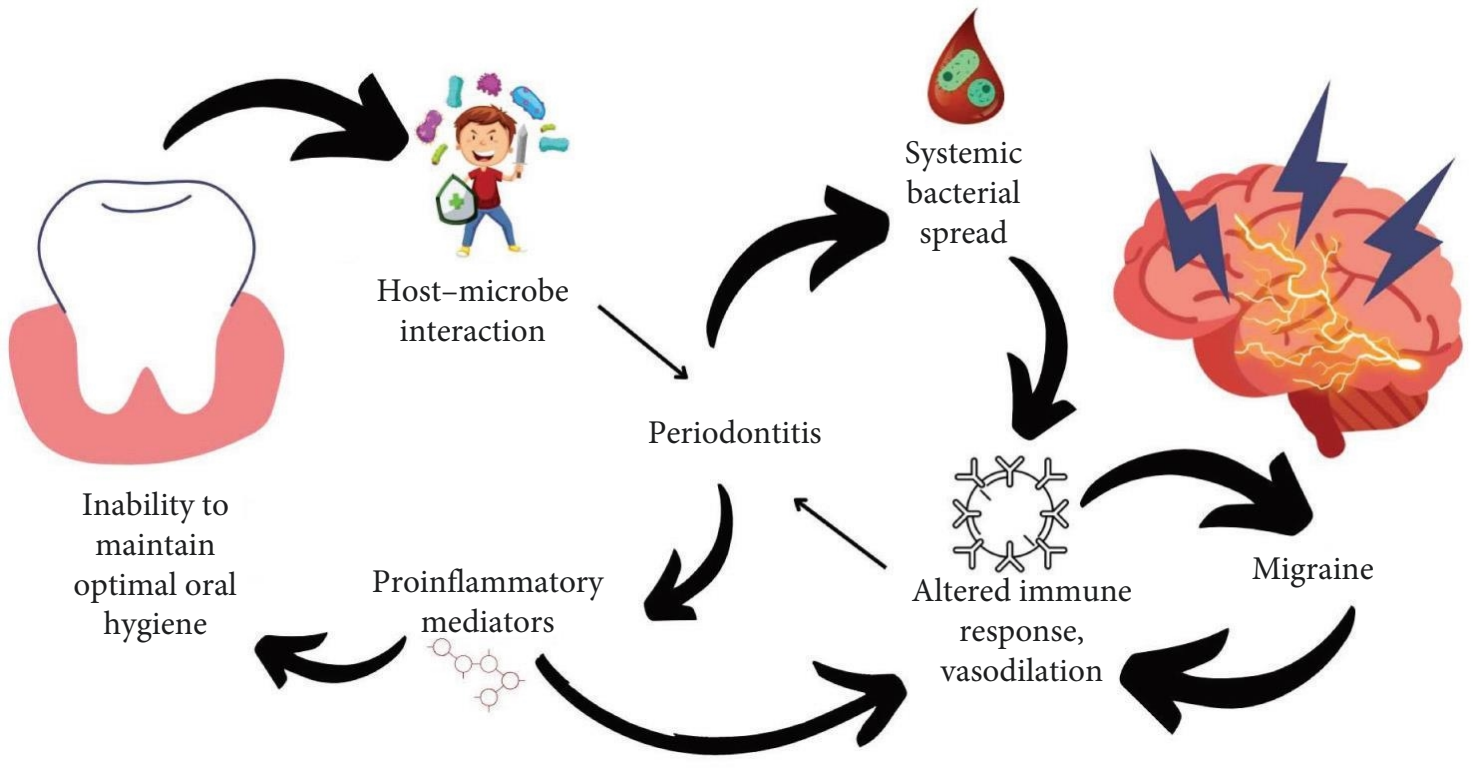
Association of poor oral hygiene, periodontitis, and migraine. Poor oral health is the key factor that precipitates the occurrence of periodontitis. The systemic spill of various direct and indirect mediators is associated with the occurrence of migraine. Inhibiting this pathophysiological link by maintaining optimal oral hygiene is the key to disrupt this oral-systemic connect.

Chronic low-grade inflammation such as periodontitis is characterized by the vascular dissemination of various chemical mediators which are either metabolized in the liver in response to the bacterial challenge or directly at the site of periodontal infection. Since, periodontitis remains untreated in most of the cases, the chemical mediators stay suspended in the blood stream thereby, placing the individual at a higher risk of systemic disease due to an increase in the inflammatory portfolio [16]. Apart from the direct bacterial spread; various indirect mechanisms that link migraine and periodontitis, which are primarily caused due to the systemic spill of these chemical mediators, is explained in this section (Figure 2).

#### 3.2.1. Proinflammatory Mediators

According to numerous studies, periodontal patients have elevated levels of systemic inflammatory mediators than healthy controls, including higher levels of C-reactive protein (CRP), Interleukin 1 (IL-1), Interleukin 6 (IL-6), and Tumor Necrosis Factor-*α* (TNF-*α*) which is also similar to the pattern of inflammatory mediator expression in patients with migraine. These cytokines have been linked to vascular dysfunction and are proinflammatory in nature. A study by Leira et al. [17], suggested that leptin and procalcitonin were promising inflammatory biomarkers which were elevated in patients with chronic periodontitis and migraine [17, 18]. It is suggested that procalcitonin causes sterile meningeal inflammation which is linked to the occurrence of migraine which further emphasizes the altered inflammatory response in migraine [19].

In addition, to being implicated in migraine pathogenesis, elevated leptin levels also contribute to the chronification of this illness through systemic inflammation [20].

Overall elevation of pro-inflammatory markers can trigger altered immune response and vasodilation within the periodontium and also cause a systemic spill of inflammation leading to neurogenic inflammation and triggering migraine.

#### 3.2.2. Neurovascular Mediators

Vasodilation and enhanced vascular permeability are two distinct mechanisms that contribute to the neurogenic inflammation in migraine which is characterized by the release of various neuropeptides, including substance P (SP), neurokinin A (NKA), and calcitonin gene-related peptide (CGRP), which modulate the molecular events of migraine [21]. During migraine there is stimulation of the trigeminal pathway, the participation of peripheral and central processes, and the existence of CGRP, a crucial mediator in the pathophysiology of migraines. Serum CGRP content rises and falls in tandem with the severity of a migraine attack. The occurrence of inflammatory periodontitis and their association with migraine are pathophysiologically related to CGRP [22, 23].

SerumTNF-related weak inducer of apoptosis (s-TWEAK) and pentraxin-3 (PTX3), which are vascular systemic inflammatory mediators have also been suggested to be higher in periodontitis patients who are chronic migraineurs [24]. It is suggested that these neuroinflammatory mediators are known to activate the trigeminovascular system which is not only associated with occurrence of migraine, but also with its chronification. A cascade of inflammatory responses characterized by erythema and hyperemia (secondary to local vasodilatation), local edema (secondary to plasma-protein extravasation), and hypersensitivity (secondary to changes in the excitability of certain sensory neurons) are triggered by these neurovascular mediators interacting with endothelial cells, mast cells, immune cells, and vascular smooth muscle. Neurogenic inflammation then ensues because of the action of these mediators which causes increased vascular permeability and vasodilatation.

#### 3.2.3. Oxidative Damage

Periodontitis is a chronic inflammatory lesion which is characterized by the activation of neutrophils and macrophages which phagocytose the pathobionts as a result of which an oxidative burst occurs [25]. This oxidative burst, is characterized by an exacerbated production of various oxygen- and nitrogen-free radicals which cannot be neutralized by physiologic antioxidant mechanisms. This results in an elevated oxidative stress not only in the periodontium, but also may have systemic repercussions which are linked to oxidative damage in diseases such as cardiovascular diseases, male infertility, and neurodegenerative disorders [26–29].

Numerous research has examined how oxidative stress contributes to migraine. Eren et al. [30] observed there was no distinction between migraineurs and healthy subjects in terms of total antioxidant status (TAS), total oxidative status (TOS), or oxidative stress index (OSI), which is comparable to that of the study by Geyik et al. [31] and Eren et al. [30]. This is in contrast to the findings of Alp et al. [32], who demonstrated that patients experiencing migraine without aura had higher levels of TOS and OSI and lower levels of TAS [32]. However, these findings are contradictory, the role of oxidative stress in the pathophysiology of migraine cannot be negated and further studies are required to establish periodontal oxidant sources to be associated with migraine. It can be understood that oxidative damage can stimulate neurons to produce CGRP and directly activate trigeminal ganglion nerve cells, vascular smooth muscle cells, vascular endothelial cells, and pain receptors. This can lead to corresponding vasodilation, which exacerbates migraine attacks.

#### 3.2.4. Genetic Polymorphism

Genetic polymorphisms, which are characterized by an exaggerated phenotypic expression of a protein due to alteration in the genetic makeup, such as in periodontitis, that have gained much of an attention as they play a role in the immunomodulation [33]. Various promising genetic alterations can be mapped to the occurrence of both periodontitis and migraine apart from being associated with common risk factors such as the proinflammatory burden, smoking, and stress [34].

Polymorphisms in the inducible nitric oxide synthetase (iNOS) gene can affect the amount of NO production in periodontal lesions. Single nucleotide polymorphisms (SNPs) in the NOS2 gene maybe related to hypertensive diseases, migraines with aura, and malignant neoplasms [35].

Serotonin and dopamine, which have both been hypothesized as neurotransmitters implicated in the pathophysiology of migraine, are thought to be affected by vitamin D release. The idea that vitamin D plays a role in migraine is supported by the discovery of vitamin D receptors (VDRs) in the hypothalamus, which is a region involved in migraine pain perception [36].

VDR gene polymorphisms and TNF-*α* gene polymorphisms have been strongly associated to the occurrence of chronic periodontitis [37, 38]. It is interesting to know that VDR gene polymorphism has been studied with migraine and susceptibility for cluster headache development due to altered clinical phenotypes in the Caucasian populations [39]. Further, Iranian patients with migraine also had TaqI and FokI VDR gene polymorphisms [40].

Similarly, the relationship between TNF-*β* gene polymorphisms and particular phenotypic characteristics of migraine has been examined by Ishii et al. [41], who observed that monoamine oxidase A T941G polymorphisms and the TNF-*β* G252A gene are linked to photophobia but not osmophobia in migraine patients [41]. A second meta-analysis of five studies on Asian population found a link between TNF-*α* and 308G/A polymorphisms with the incidence of migraine [42]. The above genetic polymorphism could collectively act upon the release of various mediators and worsen the migraine pathophysiology. This suggests that could be a possible link to genetic polymorphisms and the incidence of migraine.

#### 3.2.5. Epigenetics

Although the etiopathogenesis of migraine is not clear, an additional uncertainty factor, epigenetic regulation may have a role to play in the etiopathogenesis of the condition. Since most of the epigenetic interactions cannot be predicted precisely, there is a relatively high degree of ambiguity in associating both the multifactorial diseases—periodontitis and migraine [43–45]. However, certain epigenetic modifications such as environmental stress, proinflammatory diets, and smoking have been known to be associated with deoxyribonucleic acid (DNA) methylation and histone modification which may orchestrate not only periodontal breakdown, but also the occurrence of migraine.

Currently, epigenetic mechanisms may not be ideal to prove the cause to effect association of periodontitis and migraine and hence, further studies are required to explore the fact that epigenetics is an environmental factor that regulates the above-mentioned mechanisms or is an individual key regulating factor in the simultaneous occurrence of both the diseases.

## 4. Nature of Association—Bradford Hill Criteria

The Bradford Hill criteria of causation for periodontitis to be a causative agent for the occurrence of migraine is enlisted in Table 1 [46]. With the available studies and limited evidences, it is seen that none of the criteria are being fully satisfied to prove the causality and hence further long-term studies and case control studies with a stringent study protocol and absence of confounding variables are required to further assess the nature of association between both the diseases. Recently, Dholakia et al. [51] and Mohammed et al. [22] in systematic reviews tried to assess the association of migraine and periodontitis by studying the role of various inflammatory markers such as CGRP, IL-6 and TNF-*α* elevation. Various cross-sectional studies have been conducted and have delivered results pointing out to just mere association of both the diseases and not proving the causation. Our review is in understanding with these studies and systematic reviews. The predominant reason for not being able to show a high strength of association of both the diseases may be attributed to various biases in the studies conducted, low sample sizes, different study designs and inability to diagnose both the conditions keeping in mind stringent inclusion and exclusion criteria. Hence, future research in this field should aim at establishing similar and stringent study designs across the globe so that similar or rather more reliable results are achieved, which can be made use of to draw out more reliable conclusions on the association of migraine and periodontitis.

## 5. Effect of Periodontal Treatment on Migraine

Periodontal treatment is characterized by an overall reduction in the inflammatory portfolio which presents itself clinically as an improvement in the periodontal parameters such as a reduction in bleeding on probing and clinical attachment level gain [52]. Various mechanical and chemical methods have been employed to inhibit the plaque biofilm which has a direct impact on the reduction of proinflammatory cytokines and the elevation of proresolving mediators, however, mechanical debridement in the form of scaling and root planing is the gold standard for achieving optimal periodontal health [53]. Various studies have shown that treatment of poor oral hygiene is effective in reducing systemic inflammatory burden which would eventually render the association of anatomically distinct diseases to become insignificant if periodontal treatment is carried out effectively [54].

Treatment of periodontitis may be beneficial not only in terms of inflammatory characteristics, but also in terms of enhancing endothelium's vascular function. Additionally, it has been shown that periodontal treatment significantly lowers the levels of the migraine-causing neuropeptides such as substance P and neurokinin A, in both plasma and the gingival crevicular fluid [55, 56]. A study by Linden et al. [57], suggested that periodontal treatment effectively reduced the levels of vasoactive intestinal peptide (VIP) which is known to be involved in the pathogenesis of migraine [57].

Elevated levels of CRP have been seen in migraine patients, however, a study by Bokhari et al. [54], suggested that periodontal treatment reduced the levels of CRP thereby reducing the overall inflammatory burden, and hence it can be hypothesized that down regulation in the CRP levels may have a net positive effect on migraine treatment [54, 58].

Conversely, a common group of drug—nitrous oxide synthetase inhibitors which are widely used in the pharmacological management of migraine may also have a positive effect on the periodontal parameters as a study by Leitao et al., proved that the use of NOS inhibitors in experimental periodontitis models in rats prevented alveolar bone resorption [59].

These data may imply that there is a bidirectional link in the association of both the multifactorial diseases which however requires sound scientific evidences.

Overall, it is suggested that there exists a beneficial effect of periodontal treatment on the reduction in the severity of migraine, however, future long-term and interventional studies specifically understanding the association of periodontal therapy and migraine are required to prove the same.

## 6. Dental Treatment Protocol for Migraine

### 6.1. Known Migraineurs

Adrenaline as a part of local anesthesia and caffeinated medications to be used with caution during dental therapy as they are vasoconstrictive, especially when the migraineurs are using triptans and anti-depressants (limit the dose of epinephrine to 0.04 mg). No caution or contraindication is foreseen in migraine patients who are using analgesics, ergots, and calcium channel blockers (CCBs) [8].

Known and diagnosed migraine patients should consume prophylactic antimigraine drugs such as anticonvulsant, beta blockers, and CCBs as prescribed by the neuro physician prior to any dental therapy.

### 6.2. Suspected Migraineurs

This category must include patients with suspected migraine, newly diagnosed migraine and self-reported migraine and who are not under any medications for treatment of migraine. The dental management of these patients may include the following points:

- Dental surgeon should be able to diagnose and differentiate orofacial pains of odontogenic and nonodontogenic causes for e.g., migraine [8].
- Seek neuro physicians support for hassle-free dental management if a patient is suspected with migraine prior to commencement of treatment.
- Dental appointments should be short and stress free.
- The patient should be encouraged to have an adequate amount of sleep in the night before the treatment.
- Monitor blood pressure of the patient prior to commencement of dental procedure.
- Examination of temperomandibular joint disorders should be mandatory in all suspected patients to rule out the odontogenic precipitating factors for the occurrence of migraine.
- The use of custom fabricated oral discluding devices should be considered to relieve the clenching powers of the masticatory muscles (NTI-tss plus^TM^) (National Dentex Labs, California, USA) [60].

## 7. Conclusion

Migraine is predominantly precipitated by neurochemical imbalance however, the role of altered vascular changes and the effect of proinflammatory component cannot be negated. Periodontitis is one such low-grade inflammatory disease which may act as a triggering factor for the stimulation of vasoactive mediators that in turn may be associated with the occurrence of migraine. Hence, the management of poor periodontal health may ultimately benefit the patients systemically. However, it is imperative for the neuro physicians to be aware of this association of both the multifactorial diseases to identify and rule out periodontitis as a causative factor for the occurrence of migraine. It is suggestive that neuro physicians work in unison with dentists or periodontists in specific to identify the weightage of a periodontal etiology in the manifestation of migraine.

## Figures and Tables

**Table 1:** Nature of association of migraine and periodontitis as elicited by The Bradford Hill criteria.

Criteria	Interpretation based on available data
Strength of association	OR^*∗*^ for the strength of association between periodontitis and migraine ranges from 0.811 to 2.4 as evidenced by Davis et al.^a^, Ameijeira et al.^b^, Leira et al.^c^, This is suggestive that there is a weak strength of association.
Consistency	The results are inconsistent as Ameijeira et al.^b^, have given an OR^*∗*^ of 2.4, while other authors such as Davis et al.^a^ and Leira et al.^c^, have quoted OR^*∗*^ to be in the range of 0.811–1.100.
Specificity	The occurrence of migraine is associated with the presence of various risk factors which is similar to the multifactorial oral disease—periodontitis. Hence, it would not be appropriate to say that only periodontitis specifically causes migraine.
Temporality	Huang et al.^d^, in a Taiwanese population has noted an HR^*∗∗*^ of 1.21 which suggests the temporarily of periodontitis over migraine.
Biological gradient	Davis et al.^a^, showed that as the CAL^*∗∗∗*^ increased, there was an increase in the OR^*∗*^ of developing migraine. However, this did not achieve statistical significance, this finding highlighted the biological gradient phenomenon.
Plausibility	The plausibility of periodontal disease-causing migraine and as a disease model has been studied by Ameijeira et al.^e^. He stated that periodontal disease, a low-grade systemic inflammation, leads to higher expression of neurogenic markers due to interaction with bacteria causing endotoxemia and systemic spread of periodontitis that directly promotes the activation of the trigeminovascular system leading to migraine occurrence.
Coherence	The adjusted and unadjusted OR^*∗*^ in Leira et al.^c^, study was found to be 1.406 and 1.100, respectively. However, the adjusted OR^*∗*^ did not gain any statistical significance thereby making this study noncoherent with the findings of other studies.
Experiment	Most of the studies done to explore the association between periodontitis and migraine are all RCT^#^ and biomarker-based studies. Only one available prospective cohort study is available done by Huang et al.^d^,
Analogy	There have been no previous animal studies to examine the association between periodontitis and migraine. However, a case report by Watanabe et al.^f^, has suggested that an association exists between apical periodontitis and migraine in humans.

^*∗*^ - odds ratio,  ^*∗∗*^ - hazard ratio,  ^*∗∗∗*^ - Clinical attachment loss, ^#^ - randomized controlled trial, ^a^Davis et al. [13], ^b^Ameijeira et al. [47], ^c^Leira et al. [48], ^d^Huang et al. [49], ^e^Ameijeira et al. [21], ^f^Watanabe et al. [50].

## Data Availability

Not applicable as it is a review article.
